# Upadacitinib for Severe Refractory Hidradenitis Suppurativa After Failure of Multiple Systemic Therapies: A Case Report

**DOI:** 10.7759/cureus.109546

**Published:** 2026-05-24

**Authors:** Emanuel Chew Bonilla, Xurami Vega-Del Pilar, Sthephany Benitez Rocha, Norma Miranda Contreras, Alicia Lemini López

**Affiliations:** 1 Internal Medicine, Centro Médico Nacional Siglo XXI, Instituto Mexicano del Seguro Social, Ciudad de México, MEX; 2 Dermatology, Centro Médico Nacional Siglo XXI, Instituto Mexicano del Seguro Social, Ciudad de México, MEX

**Keywords:** hidradenitis suppurativa, hurley stage iii, jak inhibitors, refractory hidradenitis suppurativa, upadacitinib

## Abstract

Hidradenitis suppurativa (HS) is a chronic inflammatory dermatosis characterized by recurrent nodules, abscesses, draining sinus tracts, and substantial impairment in quality of life. Management of severe, refractory HS remains challenging, particularly in patients with inadequate response to systemic antibiotics and biologic therapies. Increasing evidence supports the involvement of the Janus kinase/signal transducer and activator of transcription (JAK/STAT) pathway in HS pathophysiology, highlighting JAK inhibitors as potential therapeutic alternatives.

We report the case of a 64-year-old man with severe Hurley stage III HS involving axillary, inguinal, intergluteal, abdominal, and truncal regions, with extensive inflammatory activity, chronic pain, and marked functional limitation. Relevant comorbidities included folliculitis decalvans, chronic kidney disease stage G3b, anemia, hypoalbuminemia, and malnutrition. The patient had an inadequate response to multiple prolonged systemic antibiotic regimens, adalimumab therapy for six years with subsequent secondary failure, and secukinumab, which was discontinued because of clinical worsening. Due to progressive disease and the absence of effective therapeutic alternatives, treatment with upadacitinib 15 mg once daily was initiated after careful risk-benefit assessment and informed consent. Progressive and sustained clinical improvement was observed, including a reduction in inflammatory lesions, pain, purulent drainage, and functional impairment. The International Hidradenitis Suppurativa Severity Score System (IHS4) decreased from baseline values of 127-145 points to 20 points during follow-up. Treatment was well tolerated, and no significant adverse events were documented.

This case highlights the potential role of upadacitinib as a therapeutic alternative in severe, refractory HS after failure of conventional systemic and biologic therapies and supports the need for further studies to better define the efficacy and safety of JAK inhibitors in the management of HS.

## Introduction

Hidradenitis suppurativa (HS) is a chronic inflammatory dermatosis characterized by painful nodules, abscesses, draining sinus tracts, and progressive scarring that primarily affects intertriginous areas. It is estimated to affect approximately 1% of the population and is associated with a substantial physical, socioeconomic, and psychological burden, including chronic pain, depression, social isolation, functional limitation, and marked impairment in quality of life. Severe forms of HS, particularly Hurley stage III disease, remain difficult to manage because many patients fail to achieve sustained disease control despite prolonged systemic treatment [1].

Current therapeutic strategies for moderate-to-severe HS include prolonged systemic antibiotic regimens, biologic therapies, hormonal treatment in selected patients, and surgical management. Although adalimumab remains one of the principal biologic therapies used in HS, therapeutic response is often variable, and secondary treatment failure is common during long-term follow-up. More recently, additional biologic agents targeting IL-17 pathways have demonstrated efficacy in selected patients; however, a substantial proportion of individuals with severe disease continue to experience persistent inflammatory activity and significant functional impairment despite multiple therapeutic interventions [2].

HS pathophysiology involves dysregulation of innate and adaptive immune responses with increased expression of proinflammatory cytokines. Increasing evidence suggests that inflammatory mediators involved in HS converge through the Janus kinase/signal transducer and activator of transcription (JAK/STAT) signaling pathway, supporting its role as a potential therapeutic target in refractory disease [3]. Molecular and transcriptomic studies have demonstrated activation of keratinocyte-, neutrophil-, and T lymphocyte-mediated immune responses within HS lesions, further supporting the rationale for targeted immunomodulatory therapies [4,5].

Upadacitinib is a selective JAK1 inhibitor approved for several immune-mediated inflammatory disorders, including rheumatoid arthritis, psoriatic arthritis, atopic dermatitis, ulcerative colitis, and Crohn’s disease. Emerging evidence suggests a potential role for JAK inhibitors, including upadacitinib, in moderate-to-severe HS [6,7]. Recent reports and small case series have described clinical improvement in patients with refractory HS treated with JAK inhibitors; however, available data remain limited by small sample sizes, heterogeneous patient populations, and short follow-up periods. Nevertheless, published clinical experience remains limited, particularly in patients with extensive Hurley stage III disease, multiple biologic failures, significant systemic comorbidities, and long-term follow-up demonstrating sustained clinical response. We present the case of a patient with severe, refractory Hurley stage III HS who demonstrated marked clinical improvement following treatment with upadacitinib after failure of systemic and biologic therapies.

## Case presentation

**Figure 1:**
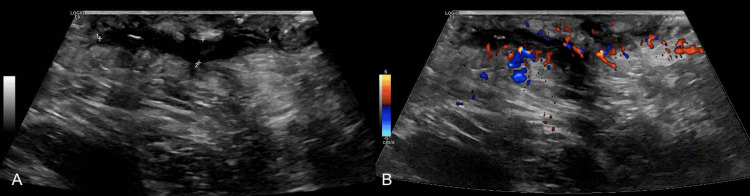
Right axillary ultrasound findings in severe hidradenitis suppurativa. (A) Gray-scale ultrasound showing an irregular subcutaneous fluid collection with internal echogenic foci and associated sinus tract formation. (B) Color Doppler image demonstrating increased peripheral vascularity consistent with active inflammation.

A 64-year-old man with severe Hurley stage III HS had been under dermatologic follow-up since 2017 because of extensive involvement of the axillary, inguinal, intergluteal, abdominal, and truncal regions. The clinical course was characterized by extensive inflammatory activity, multiple draining sinus tracts, chronic pain, continuous purulent drainage, and severe functional impairment. Ultrasound findings confirmed the diagnosis, demonstrating multiple deep inflammatory tracts (Figure 1).

Relevant comorbidities included folliculitis decalvans, phymatous rosacea previously treated with isotretinoin, major depressive disorder controlled with sertraline, chronic kidney disease stage G3b, grade III anemia likely secondary to gastrointestinal blood loss associated with prolonged nonsteroidal anti-inflammatory drug use, hypoalbuminemia, and malnutrition. These comorbidities increased therapeutic complexity and limited systemic treatment options.

Throughout the disease course, the patient received multiple prolonged systemic antibiotic regimens, including doxycycline, clindamycin, moxifloxacin, clarithromycin, and ciprofloxacin, with only partial responses and frequent relapses. Despite prolonged treatment courses, inflammatory activity persisted with progressive development of sinus tracts and scarring. The patient was evaluated by the reconstructive surgery team; however, because of the extent of disease, poor nutritional status, and unfavorable overall clinical condition, he was not considered a candidate for surgical management.

Adalimumab 40 mg subcutaneously once weekly was subsequently initiated and maintained for six years, with mild initial improvement followed by secondary loss of response. After treatment failure with adalimumab, secukinumab was initiated but discontinued after seven months because of progressive clinical worsening and persistent inflammatory activity.

**Figure 2:**
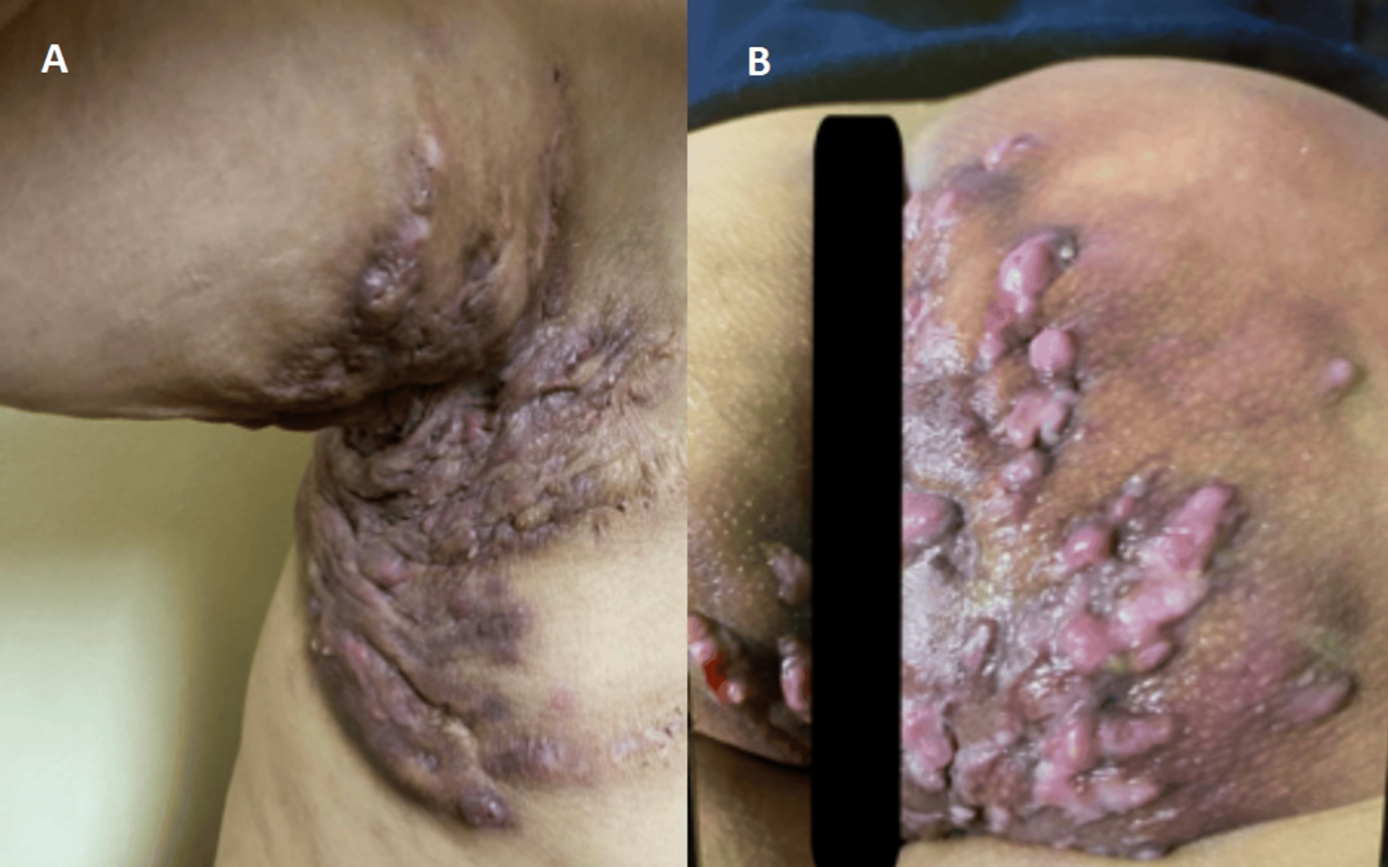
Baseline clinical presentation prior to initiation of upadacitinib therapy (Month 0). (A) Axillary region showing extensive inflammatory nodules and sinus tracts. (B) Intergluteal region with severe involvement characterized by active inflammation and draining lesions.

During the following months, the patient remained without immunomodulatory therapy and developed progressive clinical deterioration, severe functional limitation, persistent inflammatory activity, and International Hidradenitis Suppurativa Severity Score System (IHS4) values ranging from 127 to 145 points [8,9]. He also reported severe pain, continuous drainage, difficulty performing daily activities, and impaired mobility, as illustrated in Figure 2.

Given failure of multiple therapies and limited treatment alternatives, upadacitinib 15 mg once daily was initiated following a multidisciplinary risk-benefit assessment. A lower-dose regimen was selected because of the patient’s multiple systemic comorbidities, including chronic kidney disease, malnutrition, and severe anemia, in order to minimize potential adverse events while maintaining therapeutic efficacy. The off-label administration of this therapy was thoroughly discussed with the patient, and written informed consent was obtained in accordance with institutional ethical standards.

**Figure 3:**
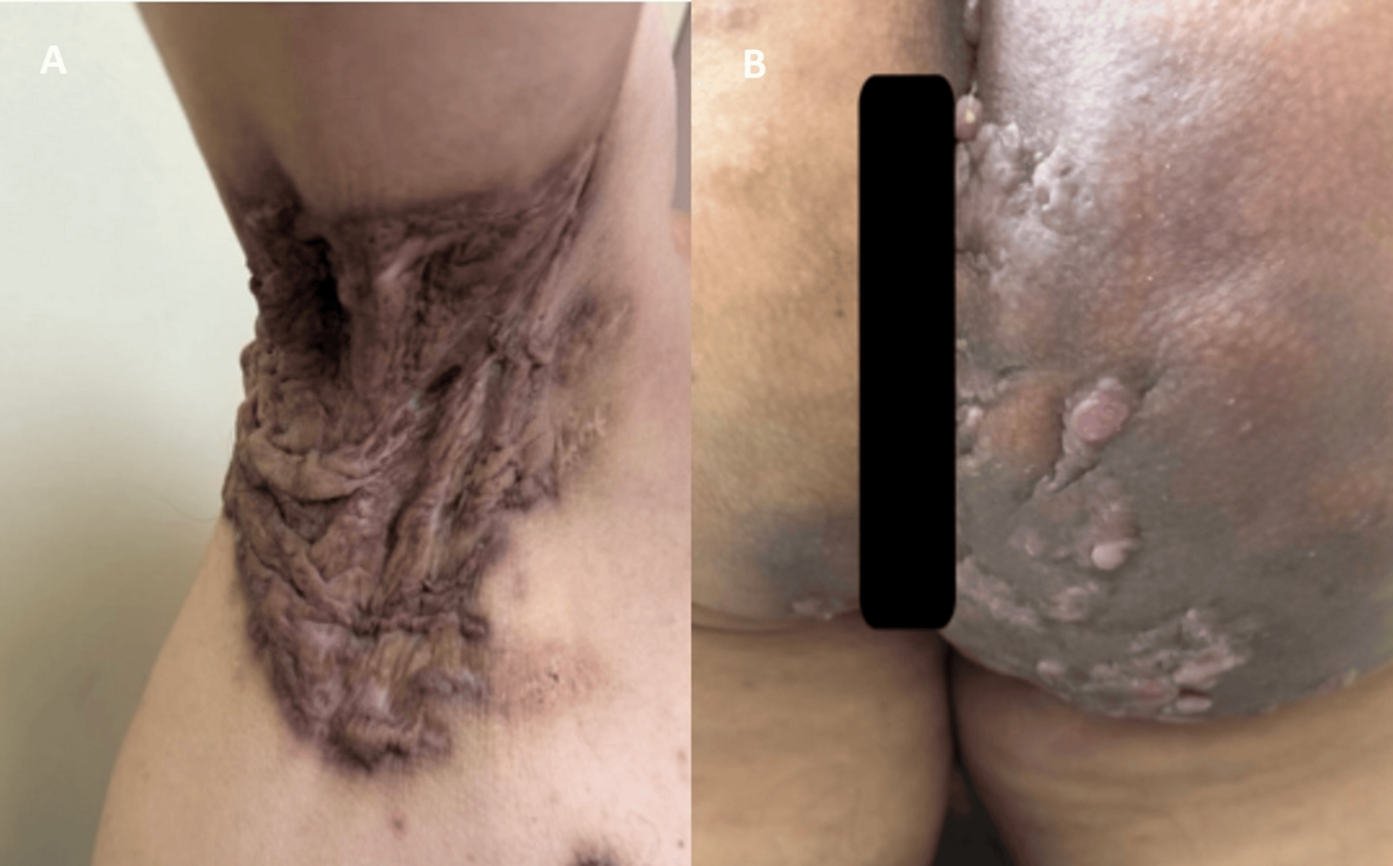
Clinical response after eight months of treatment with upadacitinib 15 mg daily (Month 8). (A) Axillary region demonstrating marked reduction in inflammatory lesions compared with baseline. (B) Intergluteal region with significant improvement and residual post-inflammatory changes.

Initial clinical improvement became evident after approximately four months of therapy, with progressive reduction in inflammatory lesions, pain, drainage, and functional limitation thereafter. During follow-up, the patient experienced an absence of new inflammatory flares and a marked reduction in pain, purulent drainage, and functional impairment. Inflammatory nodules and active draining lesions decreased substantially, as shown in Figure 3. 

The IHS4 decreased to 20 points, representing a clinically meaningful reduction from markedly elevated baseline values. Treatment was well tolerated, and no infections or clinically relevant adverse events were documented during follow-up, despite the patient’s multiple systemic comorbidities.

Comparative objective measures of disease severity and quality of life before and after upadacitinib treatment are presented in Table 1. At the time of reporting, the patient had completed eight months of follow-up with a sustained clinical response. No dose escalation was required during follow-up. Treatment was well tolerated, and no infections or clinically relevant adverse events were documented during follow-up, despite the patient’s multiple systemic comorbidities. 

**Table 1:** Baseline and follow-up disease severity and quality-of-life measures before and after upadacitinib therapy. IHS4, International Hidradenitis Suppurativa Severity Score System; DLQI, Dermatology Life Quality Index

	Before Upadacitinib	After Upadacitinib
Hurley Scale	III	III
IHS4	145	20
DLQI	26	19

**Figure 4:**
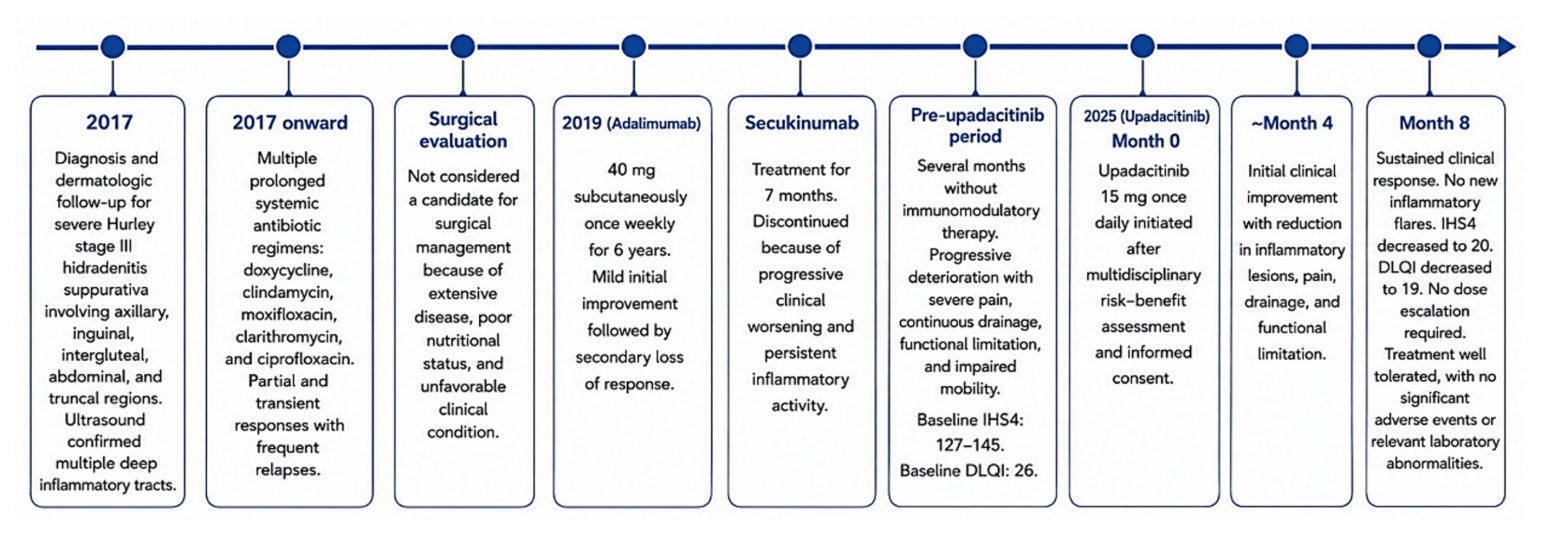
Timeline of disease course, therapeutic interventions, and clinical response to upadacitinib. IHS4, International Hidradenitis Suppurativa Severity Score System; DLQI, Dermatology Life Quality Index

No concomitant systemic corticosteroids, systemic antibiotics, or intensified wound care interventions were administered during follow-up. Although optimization of the patient’s general clinical status may have contributed partially to overall improvement, the marked and sustained reduction in inflammatory activity temporally associated with initiation of upadacitinib supports a meaningful therapeutic effect. Serial clinical and laboratory monitoring was performed throughout treatment, including complete blood count, renal function assessment, and infection surveillance. Renal function remained clinically stable, and no opportunistic infections, major hematologic abnormalities, or clinically significant laboratory alterations were observed during therapy. A chronological overview of the patient’s disease course, previous therapeutic interventions, and clinical response to upadacitinib is summarized in Figure 4.

## Discussion

HS remains one of the most therapeutically challenging inflammatory dermatoses because of its chronic, relapsing course, heterogeneous presentation, and variable response to treatment. Patients with severe Hurley stage III disease frequently experience persistent inflammation, recurrent abscesses, draining sinus tracts, extensive scarring, and major impairment in quality of life despite prolonged systemic therapy [1,2].

Increasing evidence supports the role of dysregulated cytokine signaling and JAK/STAT pathway activation in HS pathophysiology, providing a rationale for the use of JAK inhibitors in refractory disease [3-5]. In the present case, sustained clinical improvement was observed after failure of multiple systemic antibiotics and biologic therapies, including adalimumab and secukinumab, supporting the potential role of upadacitinib as an alternative therapeutic option in highly refractory HS.

Despite available antibiotic, biologic, and surgical approaches, many patients with advanced HS fail to achieve sustained disease control. Although adalimumab has demonstrated efficacy in many patients, therapeutic response is often incomplete, and secondary loss of efficacy may occur during long-term treatment. Similarly, IL-17 inhibition has emerged as an additional therapeutic strategy; however, outcomes remain heterogeneous, particularly in patients with advanced disease and extensive inflammatory burden [2].

Our patient exhibited several unfavorable prognostic factors associated with severe, refractory disease, including longstanding Hurley stage III HS, extensive anatomical involvement, multiple draining sinus tracts, markedly elevated inflammatory burden, and significant systemic comorbidities. Despite multiple prolonged systemic antibiotic regimens and biologic therapies, inflammatory activity remained uncontrolled. Additionally, the patient was not considered a candidate for reconstructive surgical management because of extensive disease and poor overall clinical condition. These factors limited therapeutic options.

The IHS4 values observed in our patient before initiation of upadacitinib therapy ranged from 127 to 145 points, reflecting extremely severe, uncontrolled inflammatory disease [8]. Following initiation of upadacitinib 15 mg once daily, the patient demonstrated progressive and sustained clinical improvement, including substantial reduction in inflammatory lesions, purulent drainage, pain, and functional limitation. The reduction of the IHS4 score to 20 points represented a clinically meaningful response, considering the severity and chronicity of disease at baseline.

Recent evidence has increasingly supported the potential role of JAK inhibitors in HS management. Several studies have explored the efficacy of JAK1 inhibition in moderate-to-severe HS, including phase II clinical trials evaluating selective JAK inhibitors [7,9]. Ackerman et al. reported favorable outcomes with upadacitinib in patients with moderate-to-severe HS in a randomized, placebo-controlled phase II study, supporting the hypothesis that selective JAK1 inhibition may reduce inflammatory activity in HS [7]. Additionally, systematic reviews have highlighted the growing interest in JAK inhibitors as therapeutic alternatives for refractory HS, particularly in patients who fail conventional systemic therapies and biologic agents [10].

Although emerging studies and early clinical trials have demonstrated promising results with JAK inhibitors in moderate-to-severe HS, published experience remains limited, particularly in patients with highly advanced and treatment-refractory disease [7,9,10]. Compared with previously reported cohorts, the present patient exhibited extensive Hurley stage III involvement, markedly elevated baseline IHS4 scores, multiple biologic failures, and significant systemic comorbidities. Despite this severe clinical profile, substantial and sustained improvement was observed during follow-up without dose escalation or major adverse events.

Published clinical experience with upadacitinib in HS remains relatively scarce. However, isolated case reports have described favorable responses in patients with refractory disease treated with selective JAK1 inhibition [11]. Our case contributes additional evidence supporting the potential efficacy of upadacitinib in severe, refractory HS, particularly in patients with extensive disease and multiple previous therapeutic failures. Notably, our patient also presented with significant systemic comorbidities and an extremely elevated baseline inflammatory burden, factors that further underscore the relevance of the observed therapeutic response.

Recent reviews have emphasized the expanding therapeutic landscape of HS and the growing role of targeted therapies, including biologics and JAK inhibitors, in difficult-to-treat disease [12].

Safety considerations remain particularly important when using JAK inhibitors, especially in patients with significant comorbidities. Upadacitinib has been associated with increased risk of infections, laboratory abnormalities, cardiovascular events, and thromboembolic complications in selected patient populations. Therefore, careful patient selection, baseline evaluation, and close clinical monitoring remain essential during therapy. In our patient, treatment was well tolerated despite the presence of chronic kidney disease, anemia, hypoalbuminemia, and malnutrition, and no serious infections or clinically relevant adverse events were documented during follow-up.

The favorable clinical response observed in this patient may be related to the broad immunomodulatory effects of selective JAK1 inhibition on multiple cytokine pathways implicated in HS pathogenesis. Several proinflammatory mediators associated with chronic HS activity, including interferons, IL-6, IL-12, and IL-23, signal through the JAK/STAT pathway. By modulating these intracellular signaling cascades, upadacitinib may reduce persistent neutrophilic and lymphocytic activity, contributing to improvement in inflammatory lesions, drainage, pain, and overall disease severity.

This case has limitations inherent to single-patient reports, including limited follow-up duration and inability to establish causal relationships or generalize outcomes to broader patient populations. Nevertheless, the substantial clinical improvement observed after failure of multiple systemic and biologic therapies suggests that upadacitinib may represent a valuable therapeutic alternative in selected patients with severe, refractory HS. Additional prospective studies and long-term real-world evidence are needed to better define the efficacy, safety profile, optimal dosing strategies, and therapeutic positioning of JAK inhibitors within the management algorithm of HS.

## Conclusions

Upadacitinib may represent a promising therapeutic alternative for patients with severe, refractory HS who fail conventional systemic and biologic therapies. In our patient, treatment with selective JAK1 inhibition was associated with marked clinical improvement, including reduction in inflammatory activity, pain, purulent drainage, and functional impairment, despite longstanding disease and multiple prior therapeutic failures. The favorable response observed in this case is consistent with emerging evidence supporting the therapeutic relevance of JAK/STAT pathway modulation in HS.

Management of advanced HS remains particularly challenging in patients with extensive anatomical involvement, severe inflammatory burden, multiple systemic comorbidities, and limited surgical or biologic treatment options. Although emerging evidence suggests that JAK inhibitors may provide clinical benefit in refractory disease, larger prospective studies and long-term follow-up data remain necessary to better define their efficacy, safety profile, and position within the therapeutic algorithm of HS.
